# Impact of Fitness Influencers on the Level of Physical Activity Performed by Instagram Users in the United States of America: Analytical Cross-Sectional Study

**DOI:** 10.3390/ijerph192114258

**Published:** 2022-11-01

**Authors:** Héctor José Tricás-Vidal, María Concepción Vidal-Peracho, María Orosia Lucha-López, César Hidalgo-García, Sofía Monti-Ballano, Sergio Márquez-Gonzalvo, José Miguel Tricás-Moreno

**Affiliations:** 1 Unidad de Investigación en Fisioterapia, Universidad de Zaragoza, Domingo Miral, s/n, 50009 Zaragoza, Spain; 2 School of Health Professions, University of Mary Hardin Baylor, 900 College St., Belton, TX 76513, USA; 3 Department of Endocrinology and Nutrition, Hospital Royo Villanova, SALUD, Barrio San Gregorio s/n, 50015 Zaragoza, Spain

**Keywords:** physical activity, sedentary behavior, social media

## Abstract

Background: Physical activity positively influences the general population’s health. Social media networks may promote changes in physical activity habits. This study aimed to analyze the relation between being encouraged to perform physical activity by fitness influencers on Instagram and the level of physical activity performed. Methods: An analytical cross-sectional study was performed in 890 United States residents with an Instagram account. The sample was made up of 79.2% females and of 75.4% millennials (born between 1981–1996). Physical activity was measured with the International Physical Activity Questionnaire. Sociodemographic characteristics were registered. Results: The percentage of females (*p* = 0.001), millennials (*p* < 0.001), students (*p* < 0.001), participants with normal BMI (*p* = 0.001), and participants performing more than 150 min per week of moderate physical activity (*p* = 0.014) was significantly higher in the group that felt encouraged to perform physical activity by the information posted by fitness influencers. This group spent a median of 2 h per week on Instagram checking for nutrition or exercise (*p* < 0.001). Conclusions: United States residents who felt encouraged to perform physical activity by fitness influencers on Instagram achieved more the World Health Organization recommendations for substantial health benefit for moderate physical activity. They were predominantly females and millennials with normal weight and spent more time on Instagram checking for nutrition or exercise.

## 1. Introduction

Physical inactivity is a global challenge, and is ranked as the fourth leading behavioral risk factor for global mortality [1]. On the contrary, physical activity has manifested as one of the most effective methods for positively influencing the health of the general population across all ages and for different population groups, for example, people suffering from cancer, hypertension, type 2 diabetes, coronary heart disease, neurological disabilities, and mental health disorders [2].

There is substantial heterogeneity in the way in which the population faces the realization of physical activity. Personal characteristics and physical and social environments are determinants of physical activity [3]. Health professionals make recommendations to their patients to promote physical activity, but populations also rely on new technologies, especially wearable devices, smartphone apps, and information found on social media networks [4] to afford the performance of physical activity. Digital interventions can motivate and stimulate individuals to promote healthy lifestyles [5]. The development of programs through social media has increased in populations such as in Australia [6], in programs such as the public and free “10,000 steps Australia Program” [7]. The study of the language and strategies used in nutrition and health accounts is increasing to favor greater engagement [8]. It has been shown that social media networks may promote changes in physical activity habits by means of social support, health education [9] and social comparison needed to facilitate behavior change [10]. Remarkably, Instagram has exponentially increased over the last decade, to the point that the 71% of adults in America have an Instagram account [11]. Engagement on Instagram in posts related to wearable devices has been found to be 30–200 times higher than on Facebook or Twitter [12]. Previous research has shown that posts soliciting feedback from participants, and counselor-initiated posts more than participant-initiated posts, favor the most engagement among participants [13]. People’s reactions to Instagrammers’ posts could serve as indicators of interest, which are precursors to intentions and the potential incorporation of better physical activity habits [12].

The majority of studies have assessed social-media-based physical activity interventions [13], but there needs to be more evidence about the influence of non-programmed interventions in changing behaviors related to physical activity in the general population, such as the influence of the information provided by fitness influencers. The potential of the information provided by fitness influencers to translate into positive behavioral change highlights a need to understand.

The aim of this study was to examine the relation between being encouraged to perform physical activity by fitness influencers on Instagram and the level of physical activity performed. Measurements of physical activity were made with the International Physical Activity Questionnaire in a sample of United States residents with an Instagram account.

## 2. Materials and Methods

### 2.1. Subjects

An analytical cross-sectional study based on self-reported and remotely collected data was performed. The study protocol was approved by the Academic Commission of the Doctoral Program in Health and Sports Sciences of the University of Zaragoza and was conducted in accordance with the ethical principles of the Declaration of Helsinki [14].

Residents in the United States of America were enrolled to participate in an anonymous online survey through an invitation via email account including a survey link to the Survey Monkey website. It was sent to former or current students from the University of Mary Hardin Baylor, The University of Kentucky, Queens University of Charlotte, and Oakland University. Additionally, the survey link was published on Instagram and Facebook. Expansion of the link was achieved with a snowball effect.

To calculate sample size, the expected proportion used was the proportion of adults in the United States of America who have an Instagram account (71%) [11]. Assuming a desired precision of +/− 3.5 percent units and a confidence level of 0.95, we determined that we should include 646 participants. Calculation was made with the GRANMO calculator [15]. To account for the possibility of surveys with missing information, we increased that number to 898.

Participants were required to be over 18 years old and to have an Instagram account. The participants, after clicking on the link to be directed to the survey, provided informed consent.

Table 1 shows the descriptive characteristics of the sample. Women constituted 79.2% of the sample. Regarding age, 11.6% were from generation Z (born 1997–2012), 75.4% were millennials (born 1981–1996), 11.5% were from generation X (born 1965–1980) and 1.6% were boomers (born 1946–1964). With respect to BMI, 0.7% were severely underweight, 1.7% were underweight, 57.4% had normal BMI, 26.1% were overweight, and 14.2% were obese. Concerning employment status, 66.9% of the subjects were currently working and 28.5% were students. Individuals in this study had been regularly consulting Instagram for a median period of 12 months, and they had spent a median of 2 h per week on Instagram checking for nutrition or exercise. The information posted by fitness influencers encouraged 68.1% of them to perform physical activity. In regard to physical activity, a median of 240.0 min per week of vigorous physical activity and a median of 180.0 min per week of moderate physical activity were performed. Participants spent a median of 5.0 h per day in sitting.

Table 2 shows the description of the types of content posted by fitness influencers that the participants consulted (*n* = 260 posts).

### 2.2. Data Sources

The participants answered the following issues in the anonymous online survey:

- Gender: Male/female.
- Age: classified into generation Z (born 1997–2012); millennials (born 1981–1996); generation X (born 1965–1980); and boomers (born 1946–1964) [16].
- Employment status: Participants were classified into the categories of subjects currently working, subjects currently not working and students.
- Height was measured in meters and weight measured in kilograms. Body mass index (BMI) was calculated: BMI = weight (kilograms)/[height (meters)]^2^. BMI was classified according to US Centers for Disease Control and Prevention BMI guidelines [17]. BMI under 15 was considered severely underweight. Between 16 and 18.4 was considered underweight. Between 18.5 and 24.9 was considered normal. Between 25 and 29.9 was considered overweight. Above 30 was considered obese. Web-based self-reported height and weight has been considered a valid method of registering height and weight, with a moderate-to-high agreement between self-reported and measured anthropometric data [18].
- How many months the participants had been regularly consulting Instagram.
- Hours per week on Instagram checking for nutrition or exercise.
- To value the potential influence of fitness influencers on Instagram on physical activity, participants answered the following question: Has the information fitness influencers posted ever encouraged you to perform a physical activity? No/Yes.

### 2.3. Physical Activity

The self-administered International Physical Activity Questionnaire (IPAQ), short form “last 7 days” [19], was used to collect the physical activity performed by the subjects. Vigorous physical activity (min per week), moderate physical activity (min per week), and time spent sitting (hours per day) were registered. IPAQ short form has shown to be a reliable and valid tool to collect physical activity data [20].

Data for vigorous and moderate physical activity were recoded. Recodification for vigorous exercises was made according to World Health Organization recommendations for substantial health benefit: less than 75 min per week (do not meet the 75 min/week recommended guidelines in for substantial health benefits) and more than 75 min per week (do meet the 75 min/week of vigorous exercise recommended in guidelines for substantial health benefits) [2]. Recodification for moderate exercises was made according to World Health Organization recommendations for substantial health benefit: less than 150 min per week (do not meet the 150 min/week of moderate exercise recommended in guidelines for substantial health benefits) and more than 150 min per week (do meet the 150 min/week of moderate exercise recommended in guidelines for substantial health benefits) [2].

### 2.4. Statistical Analyses

Firstly, a descriptive analysis was implemented. Qualitative variables were described with the absolute frequencies and the percentages in each category. Quantitative variables were described with the median and lower quartile (Q1) and upper quartile (Q3) because quantitative variables showed a non-normal distribution tested with the Kolmogorov–Smirnov test.

Subsequently, a bivariate analysis was performed. To study the relations between variables, “Has the information fitness influencers posted ever encouraged you to perform a physical activity?” was set as the independent variable. The Chi-squared test was performed for the analysis of the associations of this variable with gender, generation, employment status, BMI, and physical activity levels (the maximum likelihood ratio Chi-squared test was designated if the data set did not satisfy the assumptions of the Chi-squared test). The Chi-squared test was used to test the independence of gender, generation, employment status, BMI, and physical activity levels with respect to the independent variable. To reject the null hypothesis, statistical significance was set at *p* < 0.05. If the null hypothesis was rejected, dependency of gender, generation, employment status, BMI, and physical activity levels on being encouraged to perform physical activity by information posted by fitness influencers was shown. The Mann–Whitney *U* test was selected to compare time spent sitting and hours per week on Instagram checking for nutrition or exercise between participants who did not felt encouraged to perform physical activity by information posted by fitness influencers with those who did.

The analysis was performed using SPSS 25.0 for Mac.

## 3. Results

The analysis of the relations between gender, generation, employment status, BMI, and physical activity levels and the variable “Has the information fitness influencers posted ever encouraged you to perform a physical activity?” are shown in Table 3. The variables of gender, generation, employment status, BMI, and moderate exercise recommended in guidelines for substantial health benefits showed a significative dependency with being encouraged to perform physical activity by fitness influencers. The percentage of females, millennials, students, participants with normal BMI, and participants performing more than 150 min per week of moderate physical activity was significantly higher in the group that felt encouraged by the information posted by fitness influencers than in the group that did not. Participants who felt encouraged by fitness influencers to perform a physical activity spent more hours per week on Instagram checking for nutrition or exercise. See Table 3 for details.

## 4. Discussion

This study has examined the relation between being encouraged to perform physical activity by fitness influencers on Instagram and the level of physical activity performed in a sample of United States residents with an Instagram account. The sample was characterized by a higher proportion of women. This data are in accordance with data in Statista [21] published in June 2022, stating that 55.1% of Instagram users in the United States were women, though in our study the proportion was even higher. Regarding age, our sample is similar to the data collected in Statista, with a larger distribution of young adults among Instagram users [22], although our sample tends to be even younger. Thus, our sample is similar to samples of previous studies, which have established that young women are the predominant users of social networks [12].

According to BMI, our sample differs from the percentages previously published by Abramowitz et al., with data from participants in the National Health and Nutrition Examination Survey [23] in the United States of America. They found lower distributions of the population in severely underweight or underweight BMI (2%) and in normal BMI (32%), and larger distributions in overweight (38%) and obese (28%). The fact that our sample has an higher proportion of active subjects than the general population in the United States of America (approximately 80% are insufficiently active) [24] might be related to this result.

The information posted by fitness influencers encouraged 68.1% of the sample to perform physical activity. Of them, 82.3% were women. Previously, it has been shown that men and women behave differently in relation to information posted on the Internet [25]. Images of healthy, unhealthy, and neutral foods posted on Instagram were perceived differently between men and women. Women were more precise in the classification of food as healthy or unhealthy, and they were more prone to consume the food if it was healthy than men [25]. On the contrary, social media use, moderated by the thin-ideal model, was related to body dissatisfaction in both genders [26]. It has been proven that social media, and in particular its images, are associated in young men with the consumption of dietary supplements and anabolic androgenic steroids [27]. Women have been shown to hope for more integrity from information posted on social media [28], which might be why, in our study, they were more prone to being encouraged to perform physical activity by fitness influencers.

In our sample, in the group that felt encouraged to perform physical activity by fitness influencers, 12.5% were from generation Z and 81.0% were millennials. There is broad evidence associating younger adults [29] and middle-aged adults [7] as respondents for social media interventions, which accords with the outcomes of our study.

With respect to the difference in the percentages of employment status between the group who felt encouraged by fitness influencers and the group who did not, the percentage of students was higher in the group who felt encouraged by fitness influencers. As in our study, social media has been previously signaled as a way to enhance the performance of physical activity in college students [30,31,32].

In the group that felt encouraged to perform physical activity by fitness influencers, 61.2% had normal BMI, 25.7% were overweight and 11.4% were obese. Other previous studies have shown the mediator effect of BMI on the influence of media content and in the ability of subjects to lose weight by exercising and dieting [33]. It has been concluded that body images on Instagram does not exert an effect on those with high BMI. This is partially in accordance with our results, where the majority of the sample that felt encouraged by fitness influencers, who may assume the role of motivators generating positive feeling [18], had normal BMI.

To be encouraged to perform a physical activity by the information posted by fitness influencers was related to performing more moderate physical activity. Social media interventions have proven to be effective to increase physical activity [34]. It has been shown that dynamic social networking sites featuring regularly updated content, as fitness influencer sites usually are, might be better to facilitate physical activity than websites with static content [35]. Our data confirm the inspiring effect of Instagram on physical activity performance. However, it does not seem that it favors intense physical activity, probably because a greater long-term sports habit conditions the performance of this type of physical activity [36], and it is not so easily modifiable.

Individuals who felt encouraged to perform physical activity by the information posted by fitness influencers had not modified their total time spent sitting. In fact, they spent more time on Instagram checking for nutrition or exercise (a median of two hours per week). Time spent sitting has been considered as an independent factor for cardiovascular disease [37], independently of moderate-to-vigorous physical activity [38], to the point that replacing three hours of sitting per day with another activity, such as standing, may be associated with a decrease of 12–18% in all-cause mortality risk [39]. It appears that the motivation generated by following fitness influencers had not had a positive effect on reducing total sedentary behavior, according to the previously observed relation between general social media use and increased total time spent sitting on non-workdays [40]. Due to the potential positive effects of interrupting prolonged sedentary behavior with standing and light-intensity physical activity [41], and the adverse health effects of sedentary screen time [42], the possible impact on sedentary behavior of the use of social media, even as a source of motivation for physical activity, should be considered in future research.

### Limitations

This study is limited by the nature of the design. It is an analytical cross-sectional study, and for this reason associations can be analyzed, but without establishing causality. Data are based on self-reports, and thus the records are sensible to possible bias. The generalization of the findings might be compromised by the non-stratified sampling method. The generalizability of the results is limited to populations that share similar characteristics with the sample. Interactions of non-registered variables, such as history of competitive sports, might have influenced the results.

## 5. Conclusions

This study has shown that United States residents with an Instagram account who felt encouraged to perform physical activity by fitness influencers on Instagram achieved more the World Health Organization recommendations for substantial health benefit for moderate physical activity. They were predominantly women and millennials with normal weight and spent more time on Instagram checking for nutrition or exercise.

## Figures and Tables

**Table 1:** Descriptive characteristics of the sample.

**Gender**	***n***	**%**
Female	705	79.2%
Male	185	20.8%
**Generation**	***n***	**%**
Generation Z (born 1997–2012)	103	11.6%
Millennials (born 1981–1996)	671	75.4%
Generation X (born 1965–1980)	102	11.5%
Boomers (born 1946–1964)	14	1.6%
**Employment**	***n***	**%**
Currently working	595	66.9
Currently not working	41	4.6
Student	254	28.5
**BMI**	***n***	**%**
Severely underweight	6	0.7%
Underweight	15	1.7%
Normal	511	57.4%
Overweight	232	26.1%
Obese	126	14.2%
**Has the information fitness influencers posted ever encouraged you to perform a physical activity?**	***n***	**%**
No	284	31.9
Yes	606	68.1
**Vigorous physical activity**	***n***	**%**
Less than 75 min per week	106	11.9
More than 75 min per week	657	73.8
**Moderate physical activity**	***n***	**%**
Less than 150 min per week	351	39.4
More than 150 min per week	392	44.0
	**Median**	**Q1–Q3**
**Months on Instagram (*n* = 788)**	12.0	5.3–36.0
**Hours per week on Instagram checking for nutrition or exercise (*n* = 684)**	2.0	1.0–3.0
**Height (meters) (*n* = 890)**	1.7	1.6–1.7
**Weight (kilograms) (*n* = 889)**	68.2	59.1–80.0
**Vigorous physical activity (min per week) (*n* = 760)**	240.0	120.0–360.0
**Moderate physical activity (min per week) (*n* = 735)**	180.0	90.0–360.0
**Time spent sitting (hours per day) (*n* = 853)**	5.0	4.0–8.0

**Table 2:** Types of contents on the information posted by the fitness influencers.

Types of Content in the Posts of the Fitness Influencers (*n* = 260)	No (%)	Yes (%)
Scientific evidence on post	97.7	2.3
Post promoted a workout routine	48.5	51.5
Post promoted a single muscle group exercise	60.4	39.6
Post selling training services	51.2	48.8
Post selling supplements	82.7	17.3
Post selling clothes	58.5	41.5

**Table 3:** Comparative analysis of the characteristics of the sample according to the variable “Has the information, fitness influencers posted, ever encouraged you to perform a physical activity?”.

	Information Fitness Influencers Posted on Instagram Encouraged You to Perform a Physical Activity		
	No	Yes	*p* Value
**Gender**			
Female	72.5%	82.3%	0.001
Male	27.5%	17.7%	
**Generation**			
Generation Z (born 1997–2012)	9.5%	12.5%	<0.001
Millennials (born 1981–1996)	63.4%	81.0%	
Generation X (born 1965–1980)	23.6%	5.8%	
Boomers (born 1946–1964)	3.5%	0.7%	
**Employment**			<0.001
Currently working	79.6%	60.9%	
Currently not working	5.3%	4.3%	
Student	15.1%	34.8%	
**BMI**			
Severely underweight	1.4%	0.3%	0.001
Underweight	2.5%	1.3%	
Normal	49.3%	61.2%	
Overweight	26.8%	25.7%	
Obese	20.1%	11.4%	
**Vigorous physical activity**			
Less than 75 min per week	18.7%	11.9%	0.338
More than 75 min per week	81.3%	88.1%	
**Moderate physical activity**			
Less than 150 min per week	50.0%	46.1%	0.014
More than 150 min per week	50.0%	53.9%	
**Time spent sitting (hours per day)**	5.0	5.0	0.100
**Hours per week on Instagram checking for nutrition or exercise**	1.0	2.0	<0.001

## Data Availability

The datasets analyzed during the current study are available from the corresponding author on reasonable request. All data analyzed during this study are included in this published article.
